# Specifics of the Molecular Conformations and Physicochemical Properties of Merocyanine Form of Spirooxazine Derivative: Insights from Experimental and Molecular Dynamics Studies

**DOI:** 10.3390/ma18112505

**Published:** 2025-05-26

**Authors:** Andreea Neacsu, Viorel Chihaia, Valentin Alexiev, Georgi B. Hadjichristov, Stela Minkovska

**Affiliations:** 1 Ilie Murgulescu Institute of Physical Chemistry, Romanian Academy, Spl. Independentei 202, 060021 Bucharest, Romania; addneacsu@icf.ro; 2 Institute of Catalysis, Bulgarian Academy of Sciences, Acad. G. Bonchev Str., Bl. 11, BG-1113 Sofia, Bulgaria; valex000@gmail.com; 3 Georgi Nadjakov Institute of Solid State Physics, Bulgarian Academy of Sciences, 72 Tzarigradsko Chaussee Blvd., BG-1784 Sofia, Bulgaria; georgibh@issp.bas.bg; 4 “National Centre of Excellence Mechatronics and Clean Technologies”, Kl. Ohridski Blvd, 8, Bl. 8, BG-1000 Sofia, Bulgaria

**Keywords:** spirooxazines, merocyanine, solvatochromic properties, molecular dynamics, solvent polarity

## Abstract

This research focuses on the merocyanine form of a new synthesized spiroindolinonaphthoxazine compound. The merocyanine molecule (abbreviated as MC) has multiple fragments with different degrees of mobility. The conformational changes and the flexibility of MC in presence and in absence of the solvent molecules were studied by Molecular Dynamics simulations, providing insights into how they orient and interact with each other and with solvent molecules. The molecular packing of MC in presence and in absence of solvents with different polarities was thoroughly investigated in order to determine how the physicochemical interactions with the solvent influence the structure and stability of the MC molecule. Furthermore, the powders of MC obtained from its solutions in water, methanol, ethanol, and acetonitrile were experimentally characterized using differential scanning calorimetry, thermogravimetry, Fourier transform infrared spectroscopy, and scanning electron microscopy. Both calculations and experimental results reveal the effect of the solvent polarity on the dissolved MC molecule.

## 1. Introduction

The color-changing phenomenon is usually related to an energy source such as an external or internal stimulus. Chromogenic materials exhibit various types of chromism, such as photochromism, electrochromism, thermochromism, mechanochromism, solvatochromism, chemochromism, and biochromism, depending on the nature of the stimulus [1]. Solvatochromism refers to the phenomenon where the color of a compound changes when dissolved in different solvents. Solvatochromism can be used to sense small concentrations of polar molecules in a non-polar system, to detect explosives [2], as well as to evaluate certain solvent parameters to assess solubility phenomena and predict suitable solvents for particular applications [3]. A water-molecule- or humidity-responsive system, called a hydrochromic material, is also used as functional inks and textile colorants in industrial systems [4]. In particular, the solvatochromic properties of merocyanine compounds make them valuable as polarity indicators for studying solvent effects on molecular structure [5].

**Figure 1:**
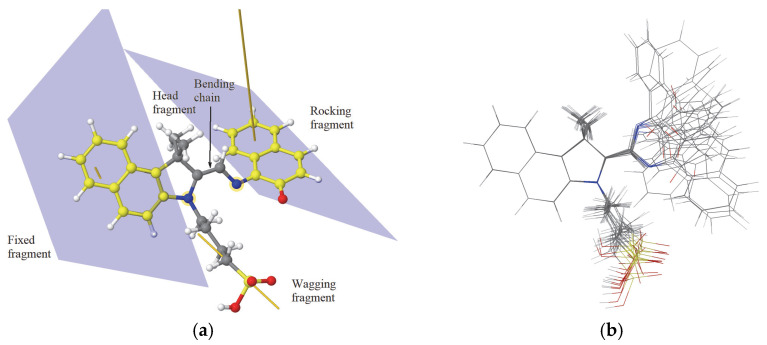
The MC functional regions (**a**) and various stable isomers with the superposed fragment C_10_NH_6_ (**b**). The carbon atoms of the two fragments C_10_NH_6_ are indicated by yellow color; the average planes that contain them are indicated, together with the corresponding normal vectors. The direction of the wagging tail is indicated by the yellow line. The colors of balls indicate the type of the respective atoms: green and gray balls—carbon, red—oxygen, blue—nitrogen, and white—hydrogen).

In this work the target compound is the merocyanine form (see Figure 1a) of a spiroindolinonaphthoxazine derivative with the corresponding IUPAC name 4-[(2E)-1,1-dimethyl-2-({[(1Z)-2-oxo-1,2-dihydronaphthalen-1-ylidene]amino}methylidene)-1H,2H,3H-benzo[e]indol-3-yl]butane-1-sulfonic, abbreviated here as MC. The investigated MC molecule is a new synthetized product by us, exhibiting photochromic and solvatochromic properties [6]. The bond-twisting mechanism involves the conversion of closed form of the *spiro*-molecule to merocyanine (open form) through a series of steps. Upon excitation by UV light, the closed form molecule undergoes a reaction where the C*_spiro_*−O bond is broken to form the colored MC isomer. This process involves the elongation of the C*_spiro_*−O distance to reach the cis-MC structure, followed by rotation around the central torsion angle to transition to the trans-MC structure [7,8]. C*_spiro_* denotes the rigid *spiro* carbon that connects the indoline moiety and the central oxazine ring. The interconversion between these two forms can be triggered by various factors such as light, heat, or the presence of specific metal ions [9,10]. The conversion from closed form to open form (MC) under UV light irradiation and different metallic ions was already discussed in our previous works [6,8].

We attempted to use several solvents to obtain a crystalline system, but these efforts were unsuccessful. This prompted us to investigate the effects of solvents on the MC system. Indeed, the MC compound studied here is sensitive to its environment, and solvent interactions can induce changes in the MC molecular conformations, resulting in different polymorphic forms. The phenomenon of solvatochromism in spirooxazines is associated with variations in solute-solvent interactions across solvents with different polarities. The solvatochromic behavior of MC can be explained by considering the variation in geometry induced by the solvent and the stabilization of energy levels introduced through solvation [11]. The MC compound showed solvatochromic properties with solvents water, acetonitrile, ethanol, and methanol. The chosen solvents have a different number of OH groups, with characteristic solvent–solvent hydrogen bonds topology and different availability/ability to form hydrogen bonds with the solvated MC molecules that might control the MC folding and stability. During solvent evaporation, the compound adopts a specific molecular arrangement. This arrangement is influenced by the solvent’s effect on molecular conformation in solution [12,13].

To our knowledge, no theoretical or experimental investigations have been conducted on MC molecules and MC-solvent systems, aside from our own characterizations [6,8]. Thus, this article presents the initial stage of the experimental and computational characterization of solid-phase MC properties. We experimentally examined the molecular structure and thermal properties of MC polymorph powders obtained after solvent evaporation using differential scanning calorimetry (DSC), thermogravimetry (TG), attenuated total reflectance Fourier transform infrared spectroscopy (ATR-FTIR), and scanning electron microscopy (SEM). To gain deeper insights into the effects of these solvents, we decided to complement the experimental study with atomistic calculations. The conformational changes and flexibility of MCs in the presence and absence of solvent molecules are investigated by Molecular Dynamics (MD). This approach allowed us to better understand the solvent-induced effects on the properties of MC in the solid phase and the challenges associated with obtaining crystalline MC. The studied MC molecule is characterized by a triple-fragment branched topology, and we proposed an analysis scheme based on this topology in order to characterize the flexibility and the packing of the molecules. This study provides insight into how MC molecules orient and interact with each other and with solvent molecules. The obtained results are useful for tuning and designing new photochromic molecules for different specific applications. The solvatochromism of these compounds makes them interesting for practical application. Controlling molecular switching opens the door to new opportunities on fundamental and applied materials science.

## 2. Materials and Methods

### 2.1. Computational Details

The MC molecule, which exhibits an asymmetric triskelion topology, consists of several distinct functional regions (Figure 1a). The central pentagonal ring C_4_N, part of the *spiro* group, serves as a connector for various parts of the molecule. Two C_10_NH_6_ groups are observed. One group is completed by a carbon atom that belongs together with its own nitrogen to the pentagonal ring C_4_N.

The C_11_NH_6_ fragment shares a carbon and a nitrogen atom with the central C4N ring, contributing to its rigidity. Therefore, we refer to the C_11_NH_6_ fragment as fixed. It will give the orientation of the MC molecule. The second group is completed by an oxygen atom. The fragment C_10_NOH_6_ is mobile as its nitrogen atom is connected by a CH_2_ group to the nitrogen atom from the pentagonal ring C_4_N. The C_10_NOH_6_ is called rocking fragment, as it is highly mobile (see Figure 1b) due to the flexibility of the C-CH_2_-N chain (called bending chain). Three dihedral angles define the spatial arrangement of the MC conformers. Another important fragment, called wagging due its high flexibility, is the butyl-1-sulfonate chain (CH_2_)_4_−SO_3_H that is connected to the nitrogen atom of the pentagonal ring C_4_N and ends with the sulfonyl group SO_3_H. The spatial configuration of the wagging (CH_2_)_4_–SO_3_H chain is governed by five dihedral angles defined along the nitrogen–carbon backbone. The C−S bond defines the orientation of the wagging fragment. The central pentagonal ring together with the two methyl groups CH_3_ connected to one of its carbon atoms form the head fragment. The head fragment moves solitarily with the fixed fragment C_11_NH_6_.

The MC molecule, with the chemical formula C_29_H_28_N_2_O_4_S, contains 64 atoms that are already at the limit of applicability of the Ab Initio electronic structure methods, making these methods prohibited for the study of the solvation effects on the MC molecule. Due to the large number of the degrees of freedom even the semiempirical methods are not applicable for such studies. The application of the empirical force fields are recommended, but the study of the MC solvation for several solvents limits the applicability of long Molecular Dynamics or Monte Carlo simulations. Therefore, in the present study we performed several molecular simulations of the stereometric configuration of MC molecule using the empirical force field COMPASS (Condensed-phase Optimized Molecular Potentials for Atomistic Simulation Studies) [14], with its original parameterization available as a library in the simulation package *LAMMPS* [15]. The three existing parameterizations of COMPASS force field, designed for lattice energy calculations on general organic molecular crystals, gives very good results for gas, liquid, and solid systems [16]. For the visualization of the molecular systems the software *jmol* was used [17].

The most stable isomers of the isolated MC molecule, determined by COMPASS geometry optimization for fixed dihedral angles along the bending chain of MC molecules, are presented in Figure 1b. We can see that indeed the fixed fragment is almost rigid, and that the rocking fragment reorients depending on the dihedral angles along the bending chain. The solvent could influence the stability of the MC molecule, with a preference for one isomer over another. The two isomers mentioned in the Introduction, cis-MC and trans-MC, are the most stable pristine conformers (see Figure S1c,d, respectively, in the Supplementary Materials). The trans-MC isomer is more stable than the cis-MC, with 3.9 and 2.7 kcal/mol as predicted by the COMPASS FF and DFT methods, respectively (see Table S1). However, other two configurations that involve the wagging fragment folding such that the terminal OH group of the sulfonic acid SO_3_H forms a hydrogen bond with the oxygen or nitrogen atom of the bending chain ring, as shown in Figure S1a,b in Supplementary Materials. The formation of hydrogen bonds by the OH group with the oxygen atom from the opened ring is more energetically favored (with 3.7 and 3.0 kcal/mol, as predicted by the COMPASS FF and DFT methods, respectively). In this arrangement, the stability surpasses that of the trans-MC isomer by 12.5 and 4.3 kcal/mol, as predicted by the COMPASS FF and DFT methods, respectively. The DFT calculation are performed with the code *orca* [18] using the hybrid exchange-correlation functional B3LYP [19] and the basis set 6–31+G(d,p) [20] in the spin unrestricted approach. The COMPASS force field reproduced the order of the stable MC isomers predicted by the DFT calculations.

The solvation was simulated by randomly packing 10 cis-MC molecules together with 100 solvent molecules X = A (acetonitrile), E (ethanol), M (methanol), and W (water), producing 7500 configurations for each solvated system. Since the cis-MC isomer is the stable isomer following the oxazine ring opening during the transition from the spiroindolinonaphthoxazine to merocyanine form, we have selected it as reference. The high computational effort due to the high number of freedom degrees limited us to moderate size systems, but large enough to have accurate statistics, relevant for many possible isomers of MC molecule. The 100 solvent molecules assure a good mobility of the MC molecules, with sufficient number solvent molecules to properly surround each of the MC molecule. The high concentration of MC molecules corresponds to MC-solvated systems closed to desolvation state. In order to let the molecules adjust their positions and orientations based on their interactions the initial system has a low density 0.6 g/cm^3^ comparing with that characteristic to each mixture (around 1.2 g/cm^3^). The obtained systems were fully optimized, considering the fractional coordinates of atoms and the simulation box parameters.

The most stable 10 configurations of each solvated system characterized by lowest COMPASS potential energy were further equilibrated by isothermal–isobaric ensemble (NPT—constant number of particles N, pressure P and temperature T) MD simulations of 5 ns, with a time step of 1.0 fs. The temperature of 300 K was controlled by Nose-Hoover thermostat (Q_ratio_ = 0.01), and the value of the pressure (0 bar) by Berendsen barostat, which assure good volume convergence. The thermostat and barostat settings were maintained for all subsequent MD simulations. The temperature and total energy converge to equilibrium values during the MD calculations and, therefore, the MC—solvent packing is considered to be achieved.

**Figure 2:**
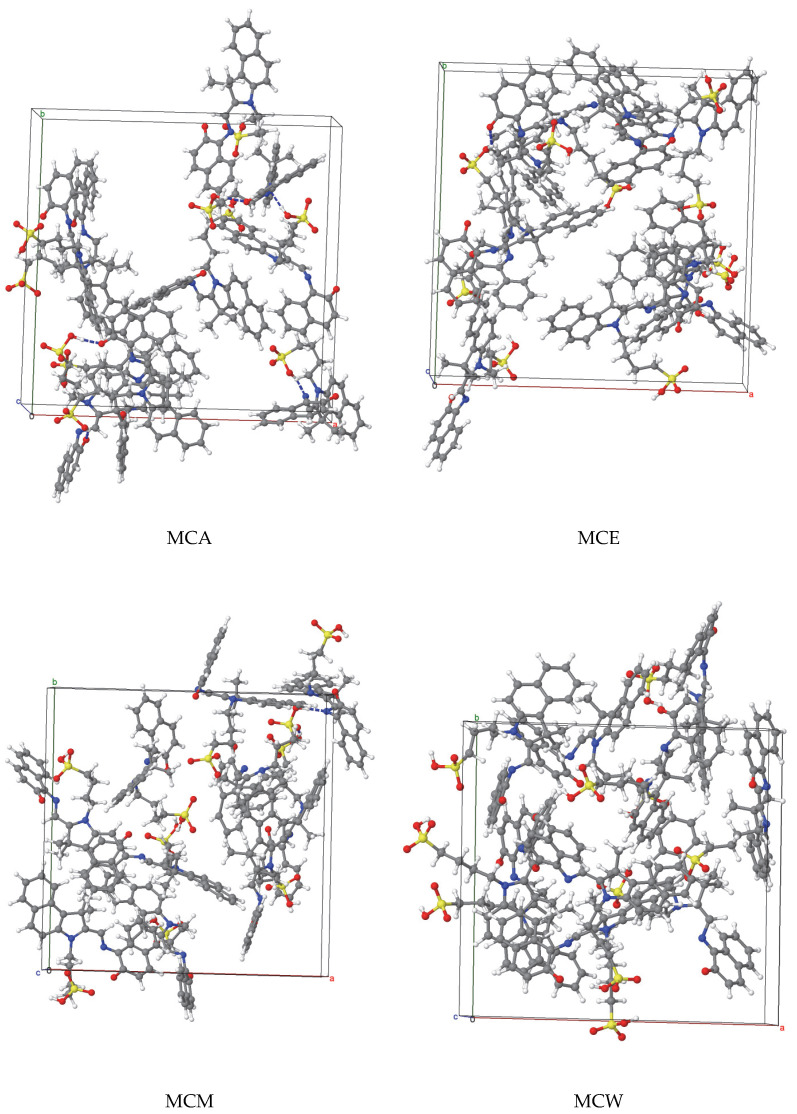
The configurations of the most stable MCX system for several solvents: acetonitrile X = A, ethanol X = E, methanol X = M and water X = W. For better visibility, the molecules of solvents are not shown. These systems are used as building units of the large 2 × 2 × 2 supercells.

The final configurations of solvated MCX (X = A, E, M, and W) were further optimized by full static calculations, for the fractional coordinates of the atoms, as well as for the size of lattice edges and for the lattice angles. The configuration characterized by the lowest potential energy for each MC system for each MCX system (see Figure 2) was selected for further relaxations by annealing for ten Molecular Dynamics—Geometry Optimization cycles, each cycle being followed by an accurate optimization step. The initial and maximum temperatures were 300 and 500 K, respectively, and the pressure was set to 0 bar. Each NPT cycle was 50 ps long.

The stabilized configurations of the ten optimized solvated MCX systems are equilibrated further MD simulations, for a fixed temperature of 300 K. We started with isothermal NVT equilibration MD simulation for each investigated MCX system. After stabilization of the system’s temperature to 300 K, the systems equilibration continued in an isothermal-isobaric NPT MD simulation, for a pressure P = 0 bar, until each system reaches a convergence for the system’s energy and density (typically around 200 ps).

The equilibrated MCX systems exhibiting the lowest average potential energy were selected for further investigation. To create larger models, each selected MCX was expanded into a 2 × 2 × 2 supercell. These larger systems, which consist of 80 MC and 800 solvent molecules, were subsequently subjected to additional equilibration using NVT for 15 ns. At the beginning of this MD stage, all replicated particles originating from the initial 1 × 1 × 1 unit cell have assigned identical velocities. However, within approximately 50 ps, their velocities became completely decorrelated (the energy deviation is reduced significantly for all systems), and by 10 ns, the periodic arrangements of particles from the original unit cells were effectively lost. This procedure enables rapid equilibration of the larger MCX systems. The MCX systems were further equilibrated by NPT MD simulations for another 1 ns. To investigate the influence of the solvent on the structure of the dry systems, desolvation was performed by removing one randomly selected solvent molecule every 250 time steps during NPT MD simulations for each solvated MCX system. We refer to the desolvated molecules as MC(X), where X denotes the solvent type: A, E, M, or W. The resulting solvated and desolvated systems were further equilibrated using 10 ns NPT molecular dynamics simulations. For improved accuracy in integrating the equations of motion, the time step was reduced to 0.5 fs during these simulations.

The equilibrated configurations of the solvated MCX and desolvated MC(X) systems are characterized by analysis of the orientation of the fixed, rocking, and wagging fragments, center-center distances of the MC molecules, the intra- and intermolecular hydrogen bonds formed by the MC molecules, which were estimated by their instant values over another 1 ns of NPT production run. The orientation of the fixed and rocking fragments are defined by the normal vectors to the best-fit plane of the two hexagonal rings formed by the carbon atoms, and the orientation of the wagging fragment is defined by the normal direction of the SO_3_ group from the terminal group C-SO_3_H of the wagging fragment. The angles between the different analyzed vectors have values between 0 and 180 degrees, being measured as shorter angles formed by the support directions of two vectors.

The formation energy of the compound MC–X = MCX or MC(X) per MC molecule, with X = acetonitrile, ethanol, methanol, and water, is determined via the following equation:

$$E_{form}=\left(E\left(MC–X\right)-n_{MC}E\left(MC\right)-n_{X}E\left(X\right)\right)/n_{MC}$$

where $E\left(MC–X\right)$, $E\left(MC\right)$ and $E\left(X\right)$ are energies of the system MC-X, MC molecule in the cis-state and the solvent molecule X, respectively. $n_{MC}=80$ specifies the number of MC molecules and $n_{X}=800or0$ is the number of X molecules in the solvated or desolvated system MC-X, respectively. The cis-MC isomer was selected as reference for the MC molecule.

The various investigated properties of the solvated and desolvated MC-systems (angle distributions, radial distribution function, number of hydrogen bonds, formation energy) are time-averaged for the 1 ns during the MD production run.

### 2.2. Experimental

#### 2.2.1. Materials

The synthesis of the spirooxazine compound and its physico-chemical properties related to photochromism, as well as its MC form were described elsewhere [6,8]. The purity of the material that was used in this study both for the analysis in solid state and in the formulation of the solutions was identified as having a purity value of 99%. The fresh synthetized dark-blue powder was used in different preparations at room temperature (25 ± 2 °C). In this study, MC of concentration 10^−4^ M in different solvents was used for preparation of the desolvated powders. Solvents used in sample preparation were acetonitrile (A), deionized double distilled water (W), ethanol (E) and methanol (M). Different forms of MC were obtained by isothermal evaporation in vacuum of the solvents and the resulting powders were further analyzed by different methods.

#### 2.2.2. Methods

Thermogravimetric data (TG/dTG) and also DSC curves of MC powders were obtained using Setaram Setsys Evolution 17 (Setaram, Caluire-et-Cuire, France), with open alumina crucibles of 100 µL volume. The calorimeter was calibrated using recommended standards of indium (Δ*H_fus_* = 28.46 J/g). The calorimeter was operated at a heating rate of 10 °C/min in the temperature range from 20 °C to 600 °C. The sample mass was 1.13 mg and was scanned in flowing argon atmosphere (16 mL/min). The relative uncertainty of the measurement was ±0.15%.

Fourier transform infrared (FT-IR) spectra of the samples were recorded at room temperature by Nicolet iS10 FT-IR spectrometer (Thermo Electron Scientific Instruments LLC, Madison, WI, USA) covering the range from 4000 to 600 cm^−1^. The spectra were acquired with an average of 32 scans, with a spectral resolution of 4 cm^−1^, in attenuated total reflectance (ATR) mode.

The morphology of the samples was investigated by scanning electron microscopy (SEM) using a high-resolution microscope, FEI Quanta 3D FEG (FEI Company, Hillsboro, OR, USA) operating at 15 kV, in low vacuum mode with low vacuum secondary electron detector. The material understudy was positioned on a double-sided carbon tape, without coating.

## 3. Results

### 3.1. The MC Conformational Analysis

The stability, stereometric configuration and packing of the MC molecules are influenced by various interactions, including the electrostatic, hydrogen bonding, aromatic π-stacking, and hydrophobic and hydrophilic interactions with the neighboring MC and solvent molecules. The C_10_NH_6_ groups in both the fixed and rocking segments of the MC molecules minimize their contact with the OH- groups of the solvent molecules, significantly affecting the packing of the MC molecules. Additionally, the flexible wagging and rocking fragments can adjust their orientations to enhance their interactions with adjacent MC and solvent molecules.

The time averaged distribution of angles between the normal vectors to the average planes defined by the fixed and rocking fragments of MC molecules reveals key insights into their conformational behavior across different environments (Figure S2, Supplementary Materials). In the acetonitrile-solvated system (MCA), the angular distribution is broad, with a moderate preference centered around 90–120°, indicating substantial orientational flexibility of the rocking fragment relative to the fixed one. Upon desolvation (MC(A)), this distribution remains broad, but develops more distinct peaks, suggesting the emergence of multiple preferred conformations, due to the increased relevance of inter-MC interactions in the absence of solvent screening. Acetonitrile, with its low hydrogen-bonding capacity, imposes minimal conformational constraints on the MC molecules, allowing both solvated and desolvated systems to sample a wide range of rocking orientations.

The ethanol-solvated system (MCE) shows a slightly sharper distribution, with a peak near 130°, indicating a modest stabilization of the rocking fragment compared to MCA. After desolvation (MC(E)), the distribution broadens slightly while retaining its general shape, implying that ethanol imparts a degree of angular ordering, albeit without fully constraining flexibility.

In contrast, methanol (MCM) exhibits a more defined angular distribution, mostly spanning 60–130°, suggesting that methanol promotes more structured configurations than ethanol or acetonitrile. In the desolvated counterpart (MC(M)), the distribution broadens again, indicating that while methanol promotes tighter packing through its stronger polarity and smaller size, this effect is partially lost upon solvent removal.

Water has the most pronounced influence. In the solvated system (MCW), the rocking–fixed fragment angle distribution is the narrowest and right-shifted, with sharp peaks around 130–150°. This reflects the strong conformational restriction imposed by the extensive hydrogen-bonding network of water, which significantly structures the MC conformation. Even in the desolvated state (MC(W)), while the angular distribution becomes broader and less sharply peaked, it still resembles the solvated profile, indicating a lasting structural imprint from prior water interactions.

Overall, the ability of the solvent to form hydrogen bonds—ranked as water > methanol > ethanol > acetonitrile—correlates directly with its capacity to modulate the conformational landscape of the MC rocking fragment, progressively stabilizing distinct angular domains as hydrogen-bonding strength and polarity increase.

The average distributions of angles formed between the normal vector to the average plane of the fixed fragment and the orientation vector of the wagging fragment reveal that the wagging segment exhibits substantial conformational flexibility (see Figure S3 in Supplementary Materials). In all solvated systems, the angular distributions are broad, with the highest probability density centered around perpendicular orientations (~90–130°) relative to the fixed fragment. This indicates that the wagging fragment frequently adopts diverse conformations but retains the freedom to have a wide range of orientations.

In the acetonitrile-solvated system (MCA), the distribution remains broad, reflecting minimal steric or hydrogen bonding constraints. Due to acetonitrile poor donor and moderate acceptor character, its weak hydrogen-bonding capabilities result in limited interaction with the sulfonic acid or the polar linker atoms (O and N), thereby allowing greater wagging flexibility. Upon desolvation (MC(A)), the angular distribution shifts, exhibiting increased population at larger angles, suggesting that inter-MC interactions help stabilize more compact conformations of the wagging fragment in the absence of solvent.

The ethanol-solvated system (MCE) presents a more defined angular distribution, with a prominent peak near 110–140°, indicative of a preferred extended conformation. This suggests that ethanol can stabilize specific wagging orientations, likely through moderate hydrogen bonding with the sulfonic acid group. In the desolvated system (MC(E)), the distribution becomes broader and diffuse, with peaks spanning ~80–130°, reflecting an increase in conformational flexibility once ethanol is removed.

In methanol (MCM), the angular distribution is very broad (30–150°) and slightly bimodal, implying that methanol allows a diverse set of orientations due to its strong hydrogen-bonding capacity and small molecular size, which facilitates dynamic reorientation. After desolvation (MC(M)), the distribution sharpens considerably around 130°, indicating that the removal of methanol restricts the wagging motion and stabilizes a preferred orientation, likely through enhanced inter-MC packing and reduced steric competition.

The water-solvated system (MCW) displays a broad bimodal distribution with a notable peak between 110 and 130°, suggesting some degree of conformational preference imposed by water’s extensive hydrogen-bonding network. Water molecules likely form strong, directional hydrogen bonds not only with the sulfonic acid group but also with the polar O and N linker atoms, thereby partially constraining the wagging fragment in semi-extended conformations. Upon desolvation (MC(W)), this restriction is lifted, resulting in a broader and flatter distribution that indicates increased conformational freedom.

In summary, the average wagging angle distribution reflects a delicate interplay between solvent-induced hydrogen bonding and conformational deformation. Strong hydrogen-bonding solvents such as water and methanol initially stabilize specific wagging orientations, while desolvation tends to release these constraints, allowing MC molecules to explore a wider conformational landscape. Acetonitrile, by contrast, exerts minimal influence in both solvated and desolvated states, permitting maximum flexibility throughout.

The average angular distributions between the average plane normal of the rocking fragment and the orientation vector of the wagging fragment in solvated MC systems highlight solvent-specific influences on molecular conformational preferences. In acetonitrile (MCA), the angle distribution is relatively broad, though it displays a subtle bias toward a specific angular range. This suggests that while the rocking–wagging relationship retains flexibility in solution, there may be weak stabilizing interactions or packing preferences. Upon desolvation (MC(A)), the distribution sharpens significantly, with a distinct peak emerging, indicating a pronounced preference for a specific conformation. This reflects enhanced intramolecular ordering driven by the absence of competing solvent interactions.

For the ethanol-solvated system (MCE), the distribution is bimodal, with peaks clustered in two angular regions: 25–80° and 90–120°. This pattern suggests the coexistence of multiple stable rocking–wagging configurations, likely stabilized by ethanol’s moderate hydrogen-bonding interactions. Desolvation (MC(E)) causes the angular distribution to consolidate into a narrower range (55–95°), reflecting a convergence toward a more defined and stable conformation once solvent constraints are removed.

The methanol-solvated system (MCM) presents a broad and shallow angular distribution spanning approximately 60–140°, with no pronounced maxima. This suggests a high degree of rocking–wagging flexibility in solution, consistent with methanol’s strong hydrogen-bonding capacity and small size, which facilitate dynamic conformational sampling. Following desolvation (MC(M)), the distribution narrows and becomes modestly peaked, with a maximum centered around ~70°, indicating a reduction in conformational diversity and the emergence of a preferred geometry.

Among the solvated systems, water (MCW) produces the broadest range of angular distribution with five groups of peaks. This is attributed to water’s strong and dynamic hydrogen bonding with the sulfonic acid group and polar O and N linker atoms, which may temporarily disrupt intramolecular alignment. However, upon desolvation (MC(W)), the angle distribution contracts and becomes highly peaked, pointing to a rigid and well-defined geometry between the rocking and wagging fragments in the absence of water.

Overall, these results reveal a common trend: all solvents allow for significant rocking–wagging flexibility in solution, with the degree of freedom varying by solvent strength and polarity. Desolvation universally promotes angular restriction and conformational ordering, with the most pronounced shift observed in systems initially solvated with water.

### 3.2. The MC Packing Analysis

The analyses of the densities of the equilibrated solvated systems reveal an ordering that corresponds to the polarity of the solvents: MCE < MCA < MCM < MCW. Upon desolvation, the densities increase in the same order MC(E) < MC(A) < MC(W) < MC(M) (see Table 1). The radial distribution function (RDF) of the centers of the pentagonal pyrrole-type C_4_N ring of MC molecules provides insight into how the MC molecules are spatially distributed relative to one another in the presence and absence of solvent. The low limits of RDF (see Figure S5 in Supplementary Materials), which indicates the shortest distances between the centers of MC molecules (d_min_), are 3.88, 4.88, 5.13 and 5.63 Å for solvated MC molecules in MCW, MCE, MCM, and MCA, respectively, (see Table 1). The order changes for the desolvated MC systems, where the inter-MC distances are 3.88, 4.38, 4.38, and 4.88 Å, following the sequence MC(A) < MC(E) = MC(W) < MC(M). While a reduction in inter-MC distances was expected upon solvent removal, an increase was observed for MC(W).

The presence of a strong first peak of RDF denotes a strong MC—solvent interactions and the presence of some successive well determined peaks suggest the regular spatial arrangement of the solute molecules. The position of the first RDF peak, its height, and width indicate preferred intermolecular distances and degrees of structural organization or compactness. The profile of RDF for MCA has a flattened slope, with a first peak at distances of about 7.8 Å, which indicates a delayed structuring. The desolvated MC(A) exhibits a pronounced first peak at ~4.5 Å, which is significantly left-shifted compared to MCA, indicating a more defined and structured organization. The MCE presents a relatively intense RDF first-peak at about 6.13 Å, implying that ethanol molecules partially clustered the MC molecules. The desolvation affects this trend of clusterization of the MC molecules determining a flattener profile for RDF, with a first maximum at 6.88 Å. Both distributions for MCM and MC(M) are relatively broad, but MCM exhibits a modest first peak at 5.63 Å compared to the more diffused profile of MC(M) with a well determined first peak at 6.38 Å. This behavior is possibly due to methanol-induced clustering. MC(W) first peak located at ~4.88 Å, while MCW shows a first RDF peak at ~4.38 Å—the lowest among all solvated systems. The MCW shows several other distinct peaks for large distances. The RDF profiles of the investigated systems clearly show that solvent polarity and hydrogen-bonding strength govern MC molecular packing. Desolvation always results in compaction (shorter g(r) peak distance), except for methanol, which shows possible swelling or reorganization. Water stands out as the most structuring solvent, followed by ethanol, methanol, and finally acetonitrile.

We characterized the packing of MC molecules by examining the unoccupied space available near the MC molecules, known as the solvent surface free volume, which reflects the potential interaction space for solvent molecules around the MC molecules. In the case of MCA and MCM, a very small space is calculated (see Table 1). However, MCE and MCW show some empty spaces, particularly near the C_10_NH_6_ groups of the fixed and rocking fragments of MC molecules, which repel the OH- groups from ethanol and water. Analyzing the systems, we observe that the methanol molecules do not disperse between the MC molecules but instead accumulate in the pockets formed by the MC molecules. The MC(M) system remained after the methanol removal from the simulation box is the most stable desolvated system, being characterized by the lowest average formation energy (see Table 1). The formation energies reported in Table 1 reflect the contributions from van der Waals, electrostatics, H-bonding, and conformational strain. Acetonitrile and ethanol stabilize MC systems mainly through favorable packing and solvent–MC H-bonding or dipole interactions. Methanol and water, despite high H-bonding potential, show modest values of the formation energy gains due to dynamic and competing H-bonding environments. Solvation limits MC–MC interactions (higher formation energies), while desolvation release the constraints of MC fragments, but often at the expense of stability.

**Figure 3:**
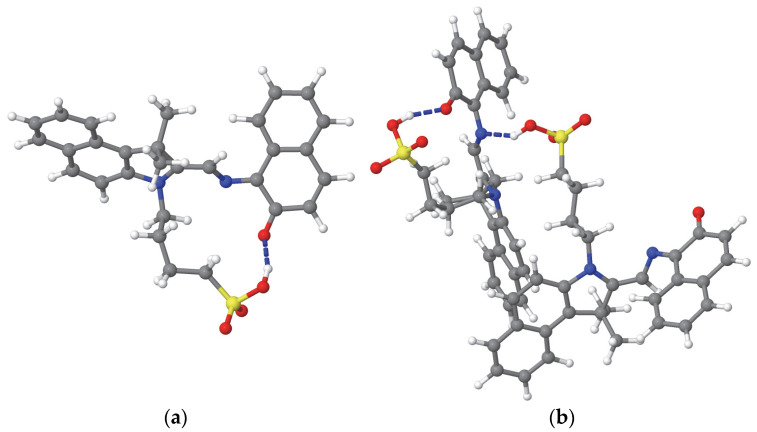
Examples of the intramolecular (**a**,**b**) and intermolecular (**b**) hydrogen bonds formed by the MC molecules solvated in acetonitrile (MCA system). The hydrogen bonds are indicated by blue dashed lines. The representation of the atoms is the same as in Figure 1.

The MC molecule has a hydrophilic sulfonic acid (–SO_3_H) terminus on a flexible wagging fragment. The nitrogen and oxygen atoms in the central region of the MC molecule can form intramolecular hydrogen bonds with the hydrogen atom of the sulfonyl group through bending of the wagging fragment within the same molecule (see Figure 3a,b) or from another MC molecule (see Figure 3b). Additionally, the sulfonic end and these central nitrogen and oxygen atoms, along with the oxygen and hydrogen atoms of the sulfonyl group, can form intermolecular hydrogen bonds with the OH groups of the solvent molecules. In contrast, the fixed and rocking fragments are hydrophobic, tending to repel the OH groups of the solvent. Table 2 presents the number of the intra-MC and inter-MC hydrogen bonds, as well as those formed with the solvent molecules. It is noted that, except for methanol, where the number of the hydrogen bonds between MC molecules remain unchanged, the number of hydrogens increase after desolvation in all other cases. The system MC(M) is characterized by a high number of intramolecular hydrogen bonds (see Table 2) that are specific to MC-HB_O_, the most stable isomer of MC. Thus, the highest stability of MC(M) compared to other desolvated systems MC(X) can be justified.

Acetonitrile, with its moderate hydrogen bond acceptor capacity, primarily forms hydrogen bonds with the sulfonic acid group of the MC molecules. In contrast, the protic solvents (ethanol, methanol, and water) engage in more extensive hydrogen bonding: they act both as hydrogen bond acceptors interacting with the sulfonic group, and as hydrogen donors forming hydrogen bonds with the sulfonic groups as well as with the central nitrogen and oxygen atoms along the MC backbone (see Table 2).

The average distributions of angles formed by normal vectors to the average planes containing the fixed fragments of neighboring MC molecules show monomodal peak around 70–100°, indicating a somewhat preferred but flexible orientation between fixed fragments (see Figure S6 in Supplementary Materials). This suggests a similar distribution pattern in angle preference, though slight variations in intensity and slight shifts between the solvated and desolvated systems. Slight flattening of the curves for MC(M) and MC(W) implies greater angular freedom. MCW shows a strongly modulated, trimodal distribution, with pronounced features at ~25°, ~82°, and ~115°, which indicates distinct preferred orientations, suggesting specific packing geometries or multi-mode interactions and possible longer-range order or hydrophobic interlocking of fixed fragments. The distribution sharpens and becomes centered near 80° by water removing, consistent with other desolvated systems. Water removal allows the MC molecules to regain intrinsic structural order, previously masked by strong solvent interactions. The presence of a low angles peak for MCE and MCW suggests the stacking of MC molecules with similar orientation of the fixed fragments.

The hydrophobic rocking fragments, being partially flexible, respond sensitively to solvent interactions and hydrogen bonding, providing insight into conformational stability and solvent-induced distortion or ordering. The rocking fragments of neighboring MC molecules in acetonitrile exhibit an average angle distribution with a fairly broad and slightly skewed profile (see Figure S7 in Supplementary Materials). The peak lies near ~90°, but the tails extend toward both lower and higher angles. The angle distribution of the desolvated MC(A) becomes narrower and more symmetrical, peaking around at 88°. This tightening suggests that in the absence of solvent, intrinsic molecular interactions stabilize as a preferred conformation. Acetonitrile allows free rotation in solutions. Upon desolvation, the MC molecules adopt a more ordered conformation dominated by intramolecular forces.

For MCE, the rocking angle average-distribution is bi-modal, with a prominent peak around 108–140°, which indicates that ethanol’s hydrogen bonding dampens rocking fragment flexibility. The angle distribution for the desolvated MC(E) becomes even narrower and more peaked, centered at ~73°, which demonstrates that ethanol pre-organizes MC conformations.

The rocking angle average-distribution for MCM is sharply defined, peaking near 85°, with narrow spread, which suggests that methanol’s strong hydrogen bonding and polarity constrain the rocking fragment efficiently. The rocking angle distribution for MC(M) is very similar to MCM, showing minimal change in angle distribution. This highlights significant pre-organization, where methanol stabilizes a geometry close to that of the desolvated system.

The MCW rocking angle average-distribution is bimodal, with fluctuations in intensity across the range, indicating a disrupted conformational behavior due to extensive hydrogen bonding with water molecules. This behavior suggests that water interacts dynamically with the rocking fragment, preventing a stable geometry from dominating. The distribution for desolvated MC(W) becomes significantly more ordered, with a strong central peak near 70°. The water molecules destabilize the rocking geometry in solution, but MC regains order in the dry state.

In conclusion, the rocking fragment is more sensitive than the fixed fragment to solvent-induced disorder or stabilization. The protic solvents like methanol and ethanol strongly restrict rocking motion, whereas aprotic acetonitrile allow high flexibility. Water causes dynamic structural noise, which resolves into ordered geometry after desolvation. These results support the idea that preorganization in solution, especially by methanol, may promote more ordered assembly in bulk phases or upon drying.

The wagging fragments of neighboring MC molecules, both in solvated and desolvated systems exhibit similar fairly broad and diffused wagging–wagging angle average-distributions, centered around 90°, with a symmetrical shape around this central peak, which correspond to a perpendicular orientation of the wagging fragments of different MC molecules. This uniform, bell-like distribution is reminiscent of an uncorrelated or weakly correlated angular relationship, typically seen in systems where interactions are non-specific or driven mainly by thermal fluctuations. This behavior highlights the notable flexibility of the wagging fragments.

### 3.3. FT-IR Analysis

**Figure 4:**
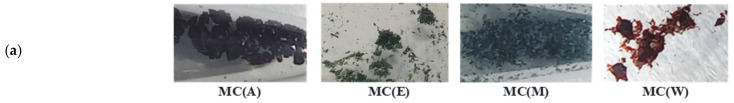

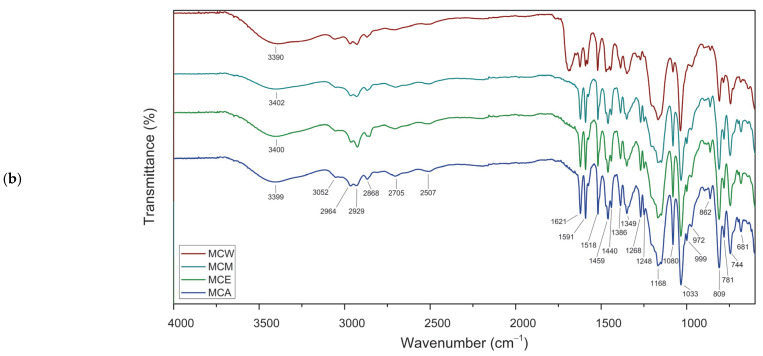
(**a**)—Photographs showing the naked-eye color of MC desolvated powders from acetonitrile—MC(A), ethanol—MC(E), methanol—MC(M), and water—MC(W*).* (**b**)—ATR FT-IR spectra for merocyanine forms MC(A), MC(E), MC(M) and MC(W).

In Figure 4a, naked-eye pictures and the color of the studied MC desolvated powders can be seen. The FTIR—ATR spectra of these powders are presented in Figure 4b and the observed vibrational frequencies of the desolvated samples are listed in Table 3. From left to right in Figure 4a, the colors of the powders range from navy to green to dark red, corresponding to the increasing polarity of the solvent. Thus, the chromatic shift in the MC polymorphs is attributed to the polarity of the solvent through solute-solvent interactions, resulting in changes such as shifts in the vibrational frequencies of certain functional groups due to modifications of bond angles and dihedral angles between atoms within the MC. By comparing the FTIR spectra of the obtained MC isomers, insignificant differences were observed for MC(A), MC(E), and MC(M), while some differences are evident for MC(W). Water provides a medium with increased hydrogen bonding capabilities; thus, the stronger interaction between water molecules and MC may result in more pronounced changes in the FTIR spectrum compared to when the molecule is in a less polar solvent environment.

For the studied samples, the associated frequencies of (C-O)*spiro* structure appear in the spectra shifted to lower wavenumbers than usual (969 cm^−1^ for MC(W) and 972 cm^−1^ for the other MC samples), which means a lengthening of the C-O bond in *spiro* structures [21]. After the ring-opening process, the spatial orientation of the butyl sulfonic moiety shown by the position of the peaks ascribed to the hydrocarbon chain (in Table 3) was affected, especially for MC(W). As shown in Figure 4, the wide characteristic band in the range of 3700 cm^−1^ to 3100 cm^−1^ (including 3399 cm^−1^, 3390 cm^−1^, 3400 cm^−1^ and 3402 cm^−1^ for MC(A), MC(W), MC(E) and MC(M), respectively) could be attributed to the –OH stretching vibration due to -SO_3_H group and of the physical adsorption of water [22].

The characteristic band at 1646 cm^−1^ only for MC(W) could be attributed to the stretching vibration of water when hydrogen bond is present. The typical band related to C=O (carbonyl stretching vibration) at 1769 cm^−1^ in MC(A), MC(E), MC(M) appear at a relatively high frequency in the IR spectra while for the MC(W) sample, the carboxyl group appears at lower frequency in IR spectrum (1765 cm^−1^). This shift occurs due to the resonance effects between the C=C or phenyl group and the C=O group, which affects the bond strength and frequency of vibration. Only in MC(W) spectrum, the pre-eminent peak at 1688 cm^−1^ and the other two faint peaks (~1765 cm^−1^ and 1646 cm^−1^) indicating the occurrence of intermolecular hydrogen bonds with a combination of phenomena related to the presence of bonded water molecules [23]. The peak at 1646 cm^−1^ in MC(W) typically could be attributed to the stretching vibration of water when a hydrogen bond is present. The specific interpretation may be related to the presence of the oxygen atom in the carbonyl group that can act as a hydrogen bond acceptor, forming hydrogen bonds with hydrogen atoms attached to electronegative atoms like oxygen or nitrogen [24,25].

In the range of 1750 cm^−1^ to 1650 cm^−1^, the spectra of the MC(A), MC(E), MC(M) isomers present a small broad peak, which arise from vibrations involving multiple bonds or a complex molecular structure caused by a possible remain solvent. The group of three separated bands at 1621 cm^−1^ (1623 cm^−1^ in MC(W)), 1591 cm^−1^ and the shoulder at 1574 cm^−1^ (1580 cm^−1^ in MC(W) are related to C=N bond and indicate that the nitrogen in the MC molecules is interacting with solute molecules [22,26]. Moreover, the presence of amide I bands (peaks at about 1621 cm^−1^ and 1623 cm^−1^) suggest the possibility of hydrogen bonds and a possible twist of naphtho-moiety of the MC molecule.

### 3.4. DSC/TG Thermal Analysis

**Figure 5:**
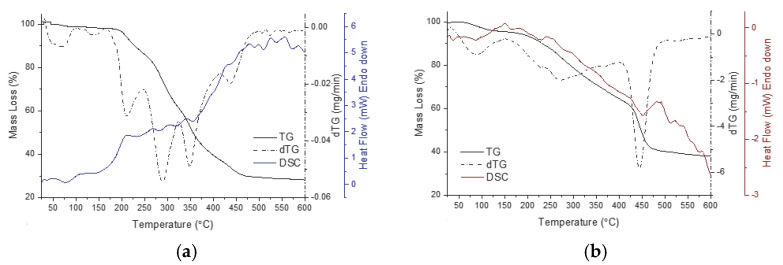

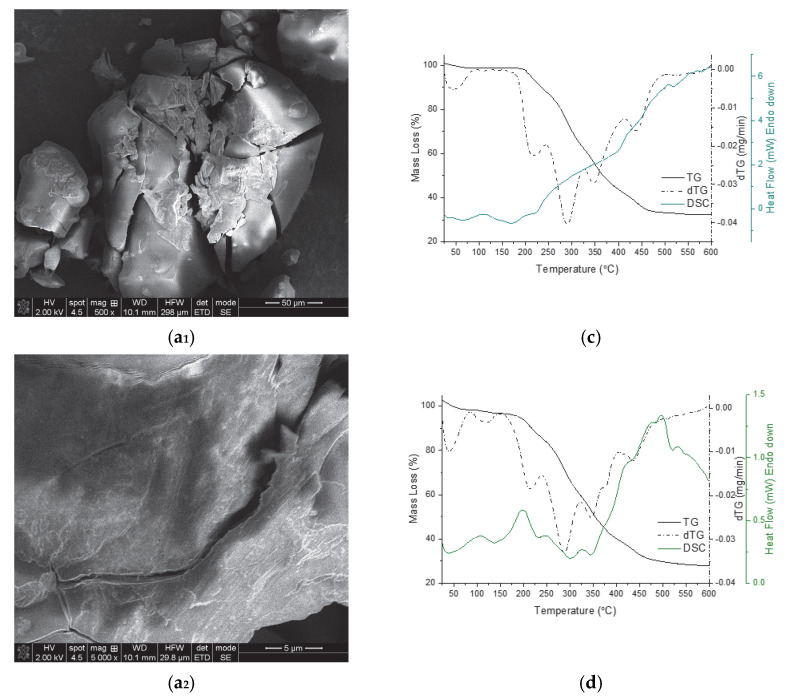
DSC (solid colored line), TG (solid black line), DTG (black dash–dot line) curves of the MC powder resulted from the desolvation of MC from acetonitrile—(**a**), water—(**b**), methanol—(**c**) and ethanol—(**d**). The pictures (**a_1_**) (magnification of 500) and (**a_2_**) (magnification of 5000) represent SEM micrographs of MC powder of sample (**a**), observed at room temperature (25 °C).

Figure 5 shows the thermal analysis results for the solid powders of the MC samples, obtained by desolvation from different solvents. The thermogravimetric parameters determined from the analysis of the thermogravimetric curves are presented in Table 4.

The DSC curves of desolvated MC powders are a complex combination of superposed thermal events. It can be seen that there are no distinctly observable phase transitions. The components resulting from the successive degradation (in steps) of the component fragments can be considered as impurities in the remaining sample mass and appear as impurities in the degradation of the following fragments of MC [27]. Thus, in the presented thermograms, enlarged endotherms can be observed for the analyzed samples. For such an endotherm, the mathematically calculated peak is very different from the observed peak [28]. MC samples present complicated thermal behavior. Therefore, the thermal stability of MC is strongly affected by its molecular conformation and by interactions with the solvent. A perspective is that the MC samples could be seen as a combination of multiple organic function groups. MC structural function groups could have their own distinct behavior concerning thermal stability. In MC, such function groups may fulfill their own function independently or in cooperation with neighboring functions. Thus, when multiple functional groups are present in a molecule, the individual thermal transitions can overlap or occur at different temperatures, resulting in multiple events on the DSC thermograms [29,30].

Another point of discussion is the polarity of the solvent, which contributes to the polymorphic selectivity of MC. This may be possible because at a molecular level the solvent selectively absorbs onto MC, respecting the presence of the function groups present in the molecule [31]. Generally, longer alkyl chains can increase the hydrophobic interactions, leading to an increase in the melting temperatures [32], but if an electrically charged group is attached to the alkyl group, the behavior upon heating will change [33,34]. Thus, the presence of an alkyl sulfate chain linked to an organic structure can affect the melting temperature by influencing the overall molecular structure of MC as well as the interactions within the MC isomer. Moreover, it enhances MC water solubility and affects the stability of merocyanine polymorph in aqueous environments [35,36]. As MC exhibited, a similar thermal behavior has been noted in the literature for ionic liquids, i.e., the melting temperature of ionic liquids with short alkyl chains decreases as the chain length increases [37]. In particular, it can be seen in Figure 5a that two solid–solid phase transitions are mainly detected in temperature ranges from 25 °C to 100 °C.

In the presented thermograms, for MC(A) the endothermic transition at about 37 °C is related to the solid–solid polymorphic transition due to the length of the hydrocarbon chain of the butyl sulfonic moiety of MC. This thermal event is followed by another transition almost without loss of mass starting at about 59 °C which corresponds to the solid–liquid phase transition of the MC. In the temperature range of 150 °C to 350 °C, the curve shows four endotherms for MC(A) due to the thermal transformations of the MC(A) fragments. The components resulting from the mass loss occurs in a wide temperature range and can be clearly observed in Figure 5a up to around the temperature of 460 °C. Table 5 presents the DSC parameters for the thermal events occurring within the temperature range corresponding to the start of heating (25 °C) until the modification of the baseline of the thermograms, over which the degradation of the samples occurs through the detachment of component fragments. The thermal events within the considered temperature range correspond to the solid–solid polymorphic transition, the solid–liquid phase transition of the MC, and the release of remaining solvent molecules, which overlap in this range. It was observed that such degradation temperature is influenced by the solvent that was used in the preparations. Further, a deconvolution of endotherms was performed in order to separate the overlapping signals of the MC and solvent located in the same temperature domain. The overlapping transitions of the heating thermograms were deconvoluted into individual constituent peaks (results are in Figure S9 and Table S2) [38]. It was observed that MC desolvates do not have a specific melting point but rather soften over a range of temperatures depending on solvent nature. The results show that the solid–solid transition at about 37 °C is clearly evidenced for MC(A) and MC(W), while for MC(M) and MC(E) samples the solvent presence slightly lower the temperature of this transition. For all the samples, a stepwise elimination of the solvent took place, possible along with another polymorphic transition. Nevertheless, several aspects, such as boiling point of the solvents and also their different abilities to form physical bonds with function groups of MC must be considered in the differentiation of such thermal behavior. In MC(A) and MC(W) samples, the solvents ethanol and methanol strongly interact with hydrophobic fragments of MC, resulting in endotherms shifting to higher temperatures in MC(M) and MC(E) samples. Considering the different hydrophobicity and hydrophilicity of the fragments of MC molecule, the obtained results suggest that methanol exhibits a balance between polar and nonpolar characteristics, thus extending the range of interactions of methanol with MC molecule more than the other solvents in this study [39]. However, in the case of the MCW sample, a particularity of the thermal behavior can be highlighted. As can be seen in Figure 5b, the rate of change in the mass of sample, as a function of temperature (dTG curves) more clearly reveals the evolution of thermal events. It is observed that the dTG curve of the MCW sample is clearly different from the other samples. Compared to the other samples, MCW shows a significant mass loss (24.61%, as shown in Table 4) at temperatures above 400 °C. This suggests that the presence of water has played a role in forming a polymorph with a more thermally resistant arrangement of MC molecules. The FTIR data and the results in Table 2 indicate that the presence of hydrogen bonds leads to stronger interactions between water molecules and MC, which may lead to more pronounced changes in desolvated MC.

In Figure 5, SEM images are shown only for the MCA sample (Figure 5a_1_,a_2_) as these were the most appropriate to present. Obtaining the SEM images was challenging due to a number of factors necessary for the accurate recording of the SEM images, which caused real-time modifications of the sample surface. Although we used reasonable recording conditions, consistent with those for sensitive organic substances, parameters such as the chamber pressure (0.08–1.5 Torr) and the energy of the electron beam used for SEM, induced changes to the sample itself (e.g., heating and charging effects), which to some extent altered the morphology during scan/imaging. However, the SEM instrument we used does not have the capability to capture high-speed events, thus limiting the ability to observe dynamic processes in sensitive samples such as MC desolvates. Nevertheless, we found that MC is highly sensitive to very fine modifications caused by different stimuli, which serves as a premise for future investigations. The fact that MC undergoes phase transitions triggered by various stimuli at physiological temperature (37 °C) makes this material ideal for biological applications.

The solvation of MC involves the interaction between the solvent molecules and its functional groups. Further, the presence of the first solvation sphere, which consists of arrangement the solvent molecules in immediate contact with the MC different function groups can affect the conformation of the MC by stabilizing certain conformations through hydrogen bonding or electrostatic interactions. The solvent polarity and its capability to form hydrogen bonds can influence the conformational preferences of the MC molecule. When the solvent molecules are removed, the intermolecular interactions between the solvent and the MC molecule are disrupted. This can lead to a change in the conformational state of the MC, as it seeks to minimize its energy and stabilize itself in the absence of solvent interactions [40,41]. It is important to note that the conformational change resulting from solvent removing is not always predictable and may vary depending on the MC—solvent system. Moreover, the Molecular Dynamics simulations suggest that solvents influence the conformation of MC molecules by causing the flexible rocking and wagging fragments to fold due to steric hindrance and hydrogen bonding. The interactions between solvent molecules, particularly through hydrogen bonds, constrain the MC molecules, reducing their spatial occupancy. Upon solvent removal, the MC molecules relax into a configuration resembling their solvated state, which in turn shapes the microstructure of the desolvated system.

The hydrogen bonding and conformational preferences of the MC molecules observed in simulations (e.g., orientation of rocking and wagging fragments, intra-/intermolecular H-bond formation, density) are supported by experimental FTIR spectra and thermal analyses (DSC/TG), which show solvent-dependent polymorphism, molecular ordering, and stability in the desolvated MC powders. The increase in hydrogen bonding upon desolvation seen in simulations is reflected in FTIR shifts associated with NH/OH groups. The conformational stabilization and packing predicted by MD (especially for methanol and water) correlate with enhanced thermal stability and specific phase transitions in DSC/TG. Solvent-induced differences in molecular arrangement in simulations match experimental observations of morphological and structural differences in the solid MC phases.

## 4. Conclusions

The integration of computational chemistry and physical property experimental evaluation can help to better characterize the merocyanine (MC) conformers of the photoresponsive spirooxazine studied here. In particular, by combining theoretical (MD calculations) and experimental (DSC, TG, SEM, and ATR-FTIR spectroscopy) results, we obtain valuable information about such MC conformers, both in solutions and as a powder. The conformational changes in the MC molecules due to desolvation of their solutions were specified as based on the impact of the interfacial environment in this case.

The MD simulations have shown the structural conformation and interaction of MC molecules with solvents—acetonitrile, ethanol, methanol, and water—as well in their desolvated state. The most stable configurations for both solvated and desolvated MC exhibit preferred orientations of flexible segments, termed “rocking” and “wagging” fragments, which favor the merocyanine isomers with intramolecular hydrogen bond. These fragments adjust their positions to maximize solvent interactions, with the C_10_NH_6_ group in MC minimizing contact with OH-groups, influencing the overall molecular packing of MC molecules. In methanol, interactions between MC molecules lead to minimal empty space due to methanol occupying pockets within MC clusters, unlike ethanol and water, which exhibit greater voids within the molecular structures. The simulations indicate that MC’s central nitrogen and oxygen atoms can form intra- and intermolecular hydrogen bonds, with the number of these bonds generally increasing upon desolvation, except in methanol. MC exhibits solvatochromism when dissolved in solvents.

The MC polymorphs obtained after desolvation were characterized experimentally. FT-IR spectral analysis was performed for the desolvated MC powders, and their thermal behavior was also discussed. It was established that the polarity of the solvent affects the polymorphic selectivity of MC by interacting with its functional groups. These interactions influence the thermal stability of MC, which reveals complex thermal events with multiple degradation steps on thermograms.

The findings of the presented study on an MC molecule self-assembly behavior in the presence and absence of various solvents (with properties influenced by the concentration and solvent polarity) indicate a competitive dynamic between intramolecular folding and intermolecular assembly, with the potential for further exploration in the development of advanced functional materials. Knowledge of how MCs solid-state structures are formed and behave can improve the stability and performance of relevant materials in practical applications.

The information obtained about the stereo-chemical configuration, the flexibility, stability, and other important physicochemical properties of the MC forms of the spirooxazine derivative considered here can be useful for a targeted preparation of such MC conformers from solutions aiming/towards their chemoresponsive functionality. In this case, the specific structure of the solid-phase MC compound that results after the evaporation of the solvent and due to the solvent’s influence on the MC molecular arrangement and stability during the transition to a MC solid state, could be very interesting for practical implications and potential applications based on the MC functional groups, orientation of the molecular fragments, molecular shapes and surfaces, molecular packing/clustering, or other possible MC constructs, e.g., composite materials and various kinds of solid (or soft) layers/films. In particular, structures containing MCs are promising to develop chemosensor systems that can be programmed to respond sensitively and specifically to targeted chemical species, such as toxic industrial chemicals, harmful heavy metal ions, or chemical warfare agents. The insights gained from this study may lead to additional innovative materials and applications such as photoactive protective coatings or films with demanded specific optical and/or barrier properties. Furthermore, MC structures can be used in the development of photocontrollable organic–inorganic hybrid materials with a significant role in nanotechnology.

Hydrogen bonding and conformational trends observed in simulations—such as rocking/wagging orientations and packing density—are directly supported by FTIR and DSC/TG experiments, which reveal solvent-dependent polymorphism and thermal stability in desolvated MC powders. Increased hydrogen bonding upon desolvation aligns with FTIR spectral shifts, and solvent-specific packing behavior seen in simulations corresponds with the thermal behavior and structure of the experimental MC phases.

## Figures and Tables

**Table 1:** The minimum distance between the MC fragments centers (d_min_), the position of the first peak of the radial distribution function (RDF), the average density, free volume, and formation energy determined by MD simulations for solvated MCX and desolvated MC(X) molecules, where X = A, E, M, W denotes the type of solvent molecules.

Solvent	State	d_min_ [Å]	First RDF Peak [Å]	Average Density [g/cm^3^]	Average Solvent Surface Free Volume [Å^3^]	Average Formation Energy per MC Molecule [kcal/mol]
Acetonitrile	MCA	5.63	7.88	1.059 (0.007)	2.38 (1.08)	−161.7 (12.7)
	MC(A)	3.88	5.13	1.220 (0.008)	37.71 (9.69)	−51.1 (8.1)
Ethanol	MCE	4.88	6.13	1.023 (0.021)	13.87 (9.91)	−153.3 (13.6)
	MC(E)	4.38	5.13	1.219 (0.005)	34.99 (9.82)	−52.3 (6.5)
Methanol	MCM	5.13	5.88	1.074 (0.005)	7.20 (2.49)	−52.5 (9.5)
	MC(M)	4.88	6.38	1.225 (0.005)	38.91 (10.31)	−52.9 (6.7)
Water	MCW	3.88	4.38	1.202 (0.005)	26.38 (8.45)	−55.6 (8.1)
	MC(W)	4.38	4.88	1.222 (0.006)	34.81 (8.01)	−43.6 (6.0)

**Table 2:** The average number and percentages of hydrogen bonds formed between different molecules for solvated MCX and desolvated MC(X) systems, where X = A, E, M, W denotes the type of solvent molecules. The values in parenthesis indicate the standard deviations.

Solvent (Solv)	State	Number of Hydrogen Bonds	Hydrogen Bonds (Donor → Acceptor) [%]				
			Intra-MC	Inter-MC	MC → Solv	Solv → MC	Solv → Solv
Acetonitrile	MCA	81.2	0.34 (0.68)	15.17 (2.77)	84.49 (2.84)	0.00	0.00
	MC(A)	85.0	18.99 (1.66)	78.62 (2.25)	-	-	-
Ethanol	MCE	128.1	0.16 (0.38)	1.81 (1.27)	7.11 (1.09)	14.70 (3.21)	76.22 (3.39)
	MC(E)	109.7	13.86 (1.97)	86.14 (1.99)	-	-	-
Methanol	MCM	1175.2	0.10 (0.05)	1.02 (0.24)	6.36 (0.27)	16.44 (0.27)	74.65 (0.82)
	MC(M)	88.1	12.71 (1.84)	87.29 (1.90)	-	-	-
Water	MCW	3350.7	0.01 (0.02)	0.84 (0.09)	2.26 (0.11)	14.24 (0.39)	82.65 (0.45)
	MC(W)	70.4	10.20 (1.29)	89.90 (1.28)	-	-	-

**Table 3:** Assignment of the ATR-FTIR peaks for MC polymorphs.

MC(A)	MC(E)	MC(M)	MC(W)	Band Assignment
3399	3400	3402	3400	O–H bending vibrations mode of H–O–H
3052	3052	3051	3055	C–H stretching of the methylene group; (sp^2^, stretch)
2964	2961	2964	2967	C–H stretching of the methyl group; (sp^3^, stretch)
2929	2925	2929	2929	C–H stretching of the methylene group; (sp^2^, stretch)
2868	2854	2868	2869	C–H stretching of the methyl group; (sp^3^, stretch)
2705	2706	2702	2709	(CO) –H stretch
2507	2506	2507	-	C–O stretching in presence of H–O–H
-	-	-	2524	C–O stretching in presence of H–O–H
1769	1769	1769	1765	C=O stretching-oxygen atom conjugated with aromatic
-	-	-	1688	C=O stretch for conjugation with aromatic double bond
-	-	-	1646	C=O bending mode in presence of H–O–H
1621	1621	1621	1623	C=O in quinone structure; amide I
1591	1591	1591	1591	–C=N– in open-chain imino; C=C in aromatic structure
1574	1574	1574	1580	C=N in aromatic structure
1518	1518	1519	1520	C=N vibrations in presence of CO
1459	1459	1459	1471	C=N vibrations; C–C in aromatic structure
1440	1440	1440	1445	C=N trans aromatic; C=C in aromatic structure; CH bend
1386	1386	1386	1386	C–H in gem-dimethyl
1349	1350	1350	1349	C–N stretching vibration
-	-	-	1288	C–N stretching vibration
1268	1269	1269	1270	C–O deformation vibration in presence of –SO_3_^−^
1248	1249	1248	1249	S–O stretching deformation in presence of C–O
1168	1168	1168	1168	C–O stretching combined with CH_3_ rocking vibration
1148	1148	1148	1148	S=O stretching in C–SO_2_–OH
1080	1080	1080	1080	–SO_3_^−^ sulfonate ion
1033	1033	1033	1036	S=O symmetric stretching in sulfonate
999	999	999	-	CH_3_ rocking vibration; –CH_2_– group
972	972	972	969	C–O stretching and C–N stretching vibrations
862	863	862	862	C–O stretching; cyclohexene derivative
809	810	810	809	aromatic C–H out-of-plane bending, –CH_2_– rocking
781	781	781	785	C–H out-of-plane; polynuclear aromatic compounds
744	745	745	743	C–C in aromatic compounds; C–N in ring
681	682	681	684	C–S stretching vibration in CH_2_–S–, C–H bending

**Table 4:** Thermogravimetric parameters (temperature domain—∆*T* and weight loss—∆*m*) of samples in temperature domain of 25 °C to 600 °C at 10 K/min.

MC(A)		MC(E)		MC(M)		MC(W)	
∆*T* [°C]	∆*m* [%]	∆*T* [°C]	∆*m* [%]	∆*T* [°C]	∆*m* [%]	∆*T* [°C]	∆*m* [%]
25–107.5	2.03	25–164.1	3.56	25–167.8	2.11	25–151.5	4.59
107.5–159.8	0.64	168.1–237.9	10.5	167.8–247.4	11.23	152.6–314.9	19.18
159.8–247.5	11.62	237.9–321.6	25.8	247.4–324.9	25.07	314.9–403.5	12.09
247.5–305.7	19.38	321.6–372.8	14.7	324.7–413.8	20.69	403.5–539.5	24.61
305.7–379.7	23.81	372.8–405.9	6.1	413.8–599.0	9.45	539.5–598.9	1.40
379.7–437.9	9.45	405.9–476.9	8.8				
437.9–599.5	5.52	476.9–599.8	2.67				

**Table 5:** Thermal parameters (the onset-, maximum-, offset-temperatures and corresponding enthalpy) of the main degradation processes of MC polymorphs from DSC data at 10 K/min.

Sample	*T*_on_ [°C]	*T*_m_ [°C]	*T*_off_ [°C]	∆*H* [J/g]
MC(A)	31.08	36.71	49.08	3.25
	51.22	79.30	107.80	30.49
	107.61	113.78	118.80	0.18
	137.36	140.87	150.22	0.70
MC(E)	22.63	38.90	109.94	45.73
	109.53	137.98	163.5	11.38
MC(M)	25.0	30.96	41.77	17.74
	45.86	67.95	96.45	27.98
	149.85	175.19	195.70	37.59
MC(W)	30.83	37.06	43.48	3.48
	68.43	85.77	98.12	6.38
	152.94	163.02	175.31	8.06

## Data Availability

The original contributions presented in this study are included in the article/Supplementary Material. Further inquiries can be directed to the corresponding authors.

## Supplementary Materials

The supporting information can be downloaded at https://www.mdpi.com/article/10.3390/ma18112505/s1, Figure S1. The most stable isomers of the MC molecule: a folded wagging fragment that forms an intramolecular hydrogen bond with either the oxygen (a) or nitrogen (b) atom from the bending chain, the trans-MC (c), and the cis-MC (d). The colors of the balls represent the atom types: gray for carbon, red for oxygen, blue for nitrogen, and white for hydrogen. The blue dashed line indicates the intramolecular hydrogen bond formed by the wagging fragment with the oxygen (a) or nitrogen (b) atom. Figure S2. The distributions of angles formed by normal vectors to the average planes that contain the fixed and rocking fragments of the MC molecules for solvated (left column) and de-solvated (right column) MC molecules (MCX—solvated MC with the solvent X = A, E, M, W, and MC(X)—de-solvated MCX). Figure S3. The distributions of angles formed by normal vectors to the average plane that contain the fixed fragment and the orientation vector of the wagging fragment of the MC molecules for solvated (left column) and de-solvated (right column) MC molecules (MCX—solvated MC with the solvent X = A, E, M, W, and MC(X)—de-solvated MCX). Figure S4. The distributions of angles formed by normal vectors to the average plane that contain the rocking fragment and the orientation vector of the wagging fragment of the MC molecules for solvated (left column) and de-solvated (right column) MC molecules (MCX—solvated MC with the solvent X = A, E, M, W, and MC(X)—de-solvated MCX). Figure S5. The Radial Distribution Functions of the centers of the MC molecules for solvated and de-solvated MC molecules (MCX—solvated MC with the solvent X = A, E, M, W, and MC(X)—de-solvated MCX). Figure S6. The distributions of angles formed by normal vectors to the average planes that contain the fixed fragments of two neighbor MC molecules for solvated (left column) and de-solvated (right column) MC molecules (MCX—solvated MC with the solvent X = A, E, M, W, and MC(X)—de-solvated MCX). Figure S7. The distributions of angles formed by normal vectors to the average planes that contain the rocking fragments of two neighbor MC molecules for solvated (left column) and de-solvated (right column) MC molecules (MCX—solvated MC with the solvent X = A, E, M, W, and MC(X)—de-solvated MCX). Figure S8. The distributions of angles formed by the orientation vectors of the wagging fragments of two neighbor MC molecules for solvated (left column) and de-solvated (right column) MC molecules (MCX—solvated MC with the solvent X = A, E, M, W, and MC(X)—de-solvated MCX). Figure S9. DSC curves for MC(A)—(a), MC(W)—(b), MCM—(c) and MC(E)—(d). The curves are de-convoluted using Gaussian distributions. The magenta line following the experimental curve (black dash-dot line) represents the fitted DSC curve. For each of the peaks, labelled as 1-4 (as the temperature increases), the temperature corresponding to the peak and fractional areas are given in Table S2. Table S1. The relative energies of some stable isomers determined by COMPASS3 force field and by DFT calculations, having as reference the energies of the most stable MC isomer MC-HB_O_. Table S2. The values and standard deviations of the temperatures T corresponding to the peaks in the DSC scans in Figure S9, as well as the corresponding fractional areas, A, for the assigned components (1–4) of the DSC curves in different MC de-solvates (from Figure S9). The coefficients of determination in all cases are *R*^2^ > 0.998.
