# Supplementary material for: Specifics of the Molecular Conformations and Physicochemical Properties of Merocyanine Form of Spirooxazine Derivative: Insights from Experimental and Molecular Dynamics Studies
Source: Materials (Basel). 2025 May 26;18(11):2505. doi: 10.3390/ma18112505 (PMC12155645; doi:10.3390/ma18112505)
Supplement: Supplementary file 1 [file materials-18-02505-s001.zip › materials-3333184-supplementary.pdf]

Supplementary Information

Specifics of the Molecular Conformations and Physicochemical Properties of Merocyanine Form of Spirooxazine Derivative: Insights from Experimental and Annealing-Molecular Dynamics Studies

Andreea Neacsu [1], Viorel Chihaia [1,*], Valentin Alexiev [2], Georgi B. Hadjichristov [3,4] and Stela Minkovska [2,4,*]

[1] Ilie Murgulescu Institute of Physical Chemistry, Romanian Academy, Spl. Independentei 202,
060021 Bucharest, Romania; addneacsu@icf.ro

[2] Institute of Catalysis, Bulgarian Academy of Sciences, Acad. G. Bonchev Str., Bl. 11, BG-1113 Sofia, Bulgaria; valex000@gmail.com

[3] Georgi Nadjakov Institute of Solid State Physics, Bulgarian Academy of Sciences, 72 Tzarigradsko Chaussee Blvd., BG-1784 Sofia, Bulgaria; georgibh@issp.bas.bg

[4] "National Centre of Excellence Mechatronics and Clean Technologies", Kl. Ohridski Blvd, 8, Bl. 8,
BG-1000 Sofia, Bulgaria

* Correspondence: vchihaia@icf.ro (V.C.); stelamin@ic.bas.bg (S.M.)

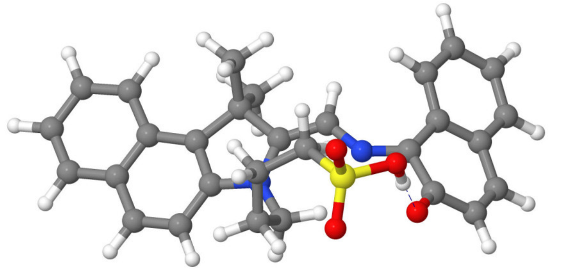

(a) - MC- HB_O

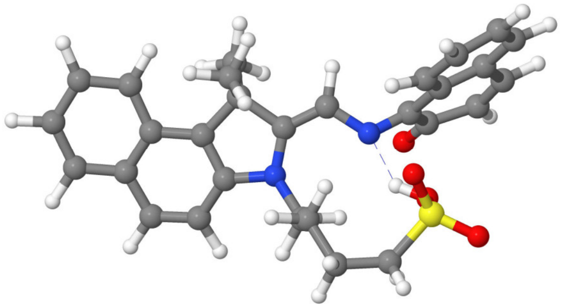

(b) - MC- HB_N

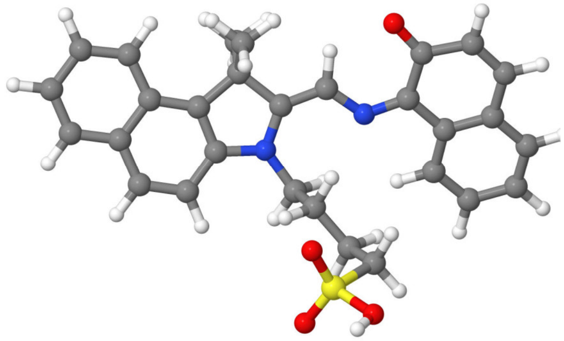

(c) - MC-trans

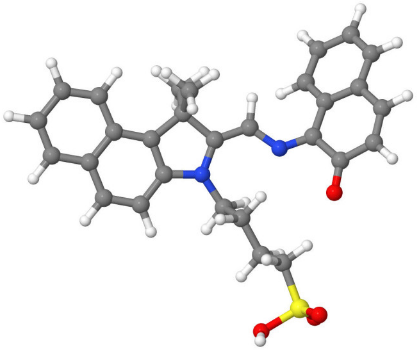

(d) - MC-cis

Figure S1. The most stable isomers of the MC molecule: a folded wagging fragment that forms an intramolecular hydrogen bond with either the oxygen (a) or nitrogen (b) atom from the bending chain, the trans-MC (c), and the cis-MC (d). The colors of the balls represent the atom types: gray for carbon, red for oxygen, blue for nitrogen, and white for hydrogen. The blue dashed line indicates the intramolecular hydrogen bond formed by the wagging fragment with the oxygen (a) or nitrogen (b) atom.

Table S1. The relative energies of some stable isomers determined by COMPASS3 force field and by DFT calculations, having as reference the energies of the most stable MC isomer MC- HB_O.

MC isomer	Relative energy [kcal/mol]	
	COMPASS3-FF	DFT (B3LYP/
MC- HB_N	3.7	3.0
MC- trans	12.5	4.3
MC-cis	16.4	7.0

Fixed - Rocking

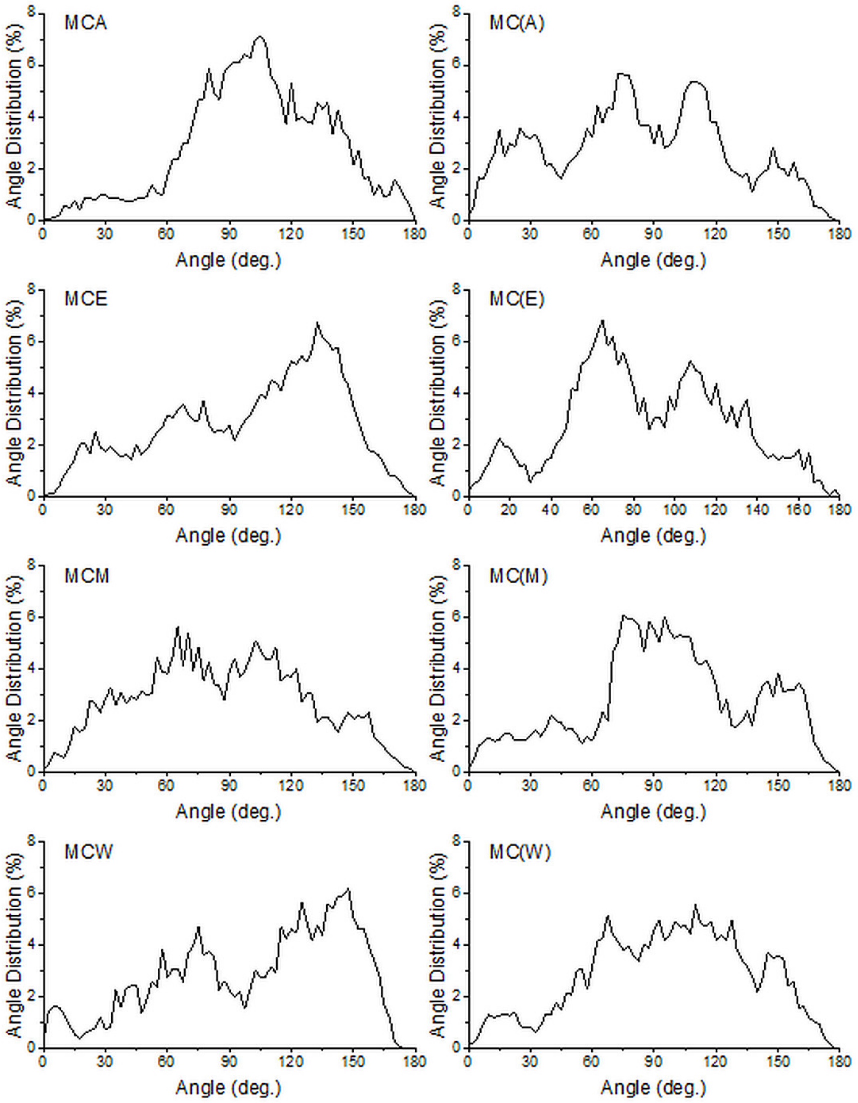

Figure S2. The distributions of angles formed by normal vectors to the average planes that contain the fixed and rocking fragments of the MC molecules for solvated (left column) and de-solvated (right column) MC molecules (MCX - solvated MC with the solvent X=A, E, M, W, and MC(X) – de-solvated MCX).

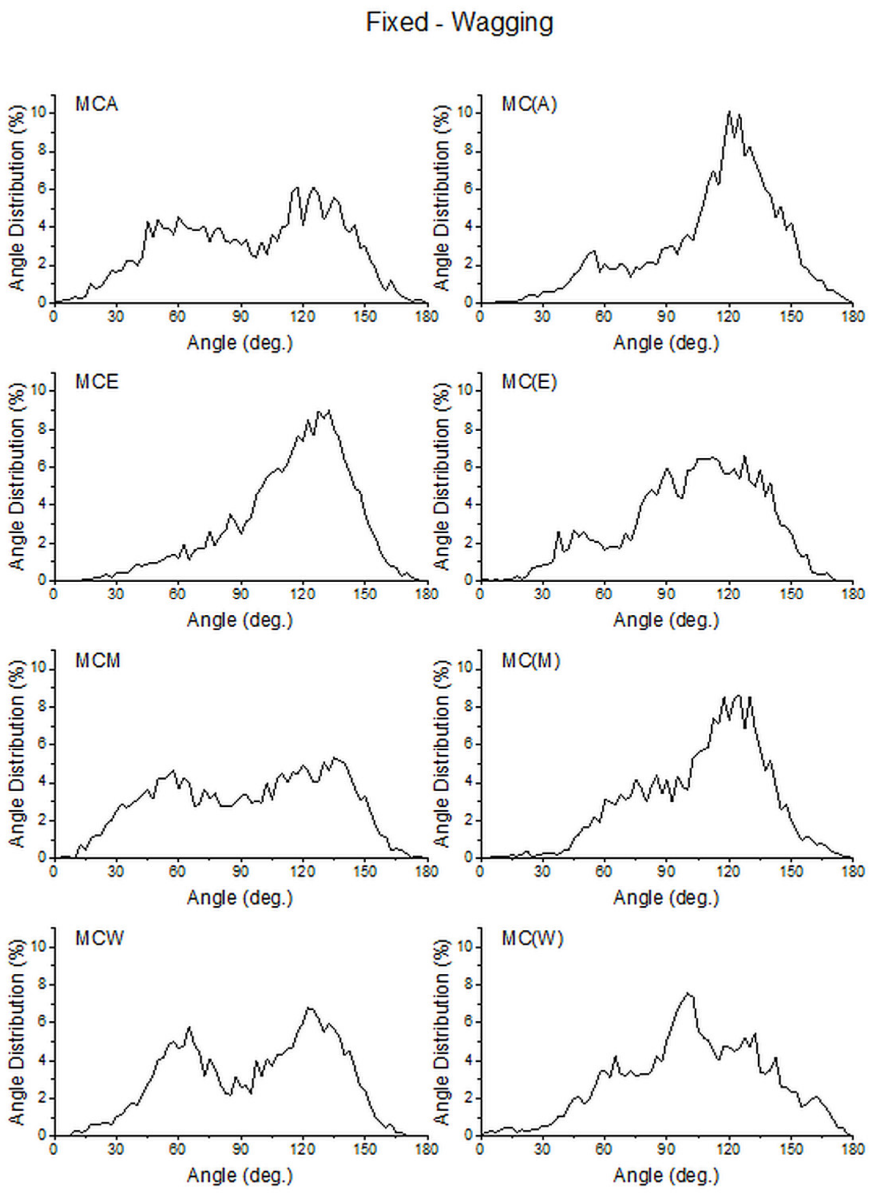

Figure S3. The distributions of angles formed by normal vectors to the average plane that contain the fixed fragment and the orientation vector of the wagging fragment of the MC molecules for solvated (left column) and de-solvated (right column) MC molecules (MCX - solvated MC with the solvent X=A, E, M, W, and MC(X) – de-solvated MCX).

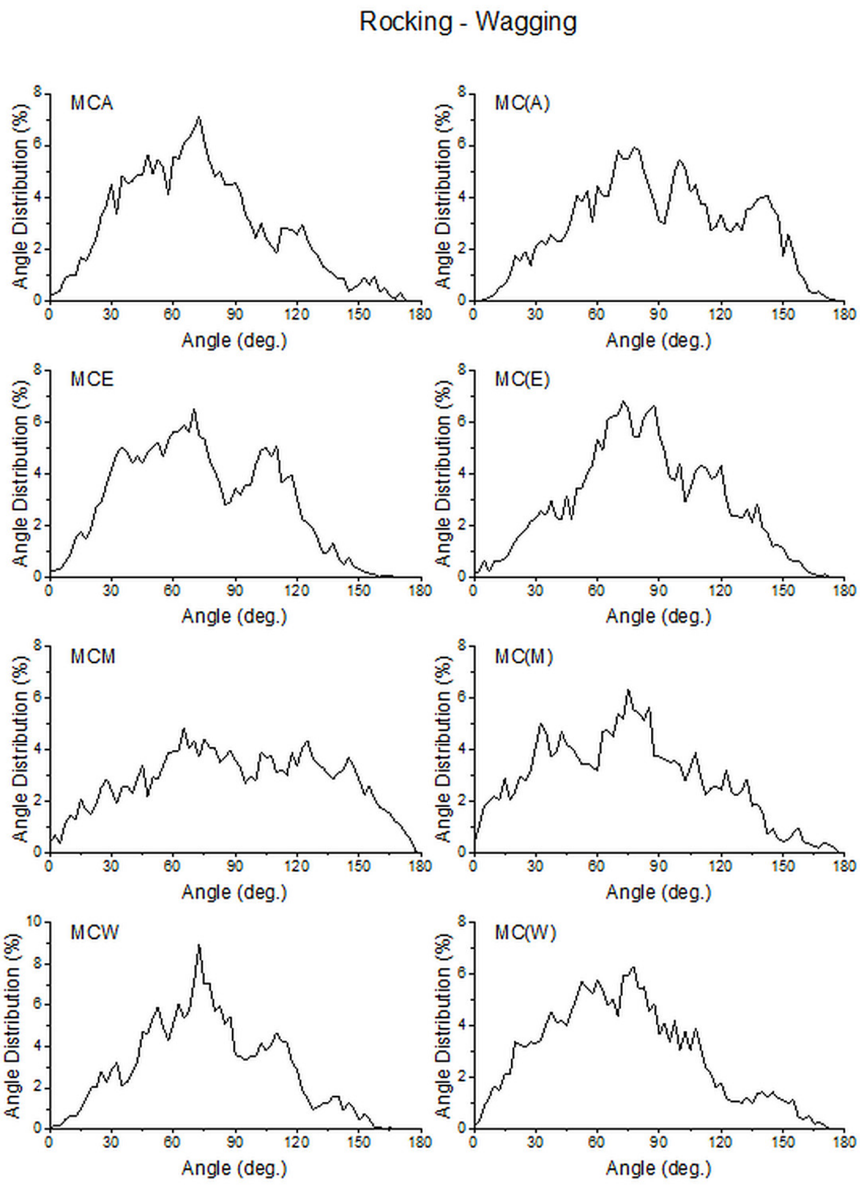

Figure S4. The distributions of angles formed by normal vectors to the average plane that contain the rocking fragment and the orientation vector of the wagging fragment of the MC molecules for solvated (left column) and de-solvated (right column) MC molecules (MCX - solvated MC with the solvent X=A, E, M, W, and MC(X) – de-solvated MCX).

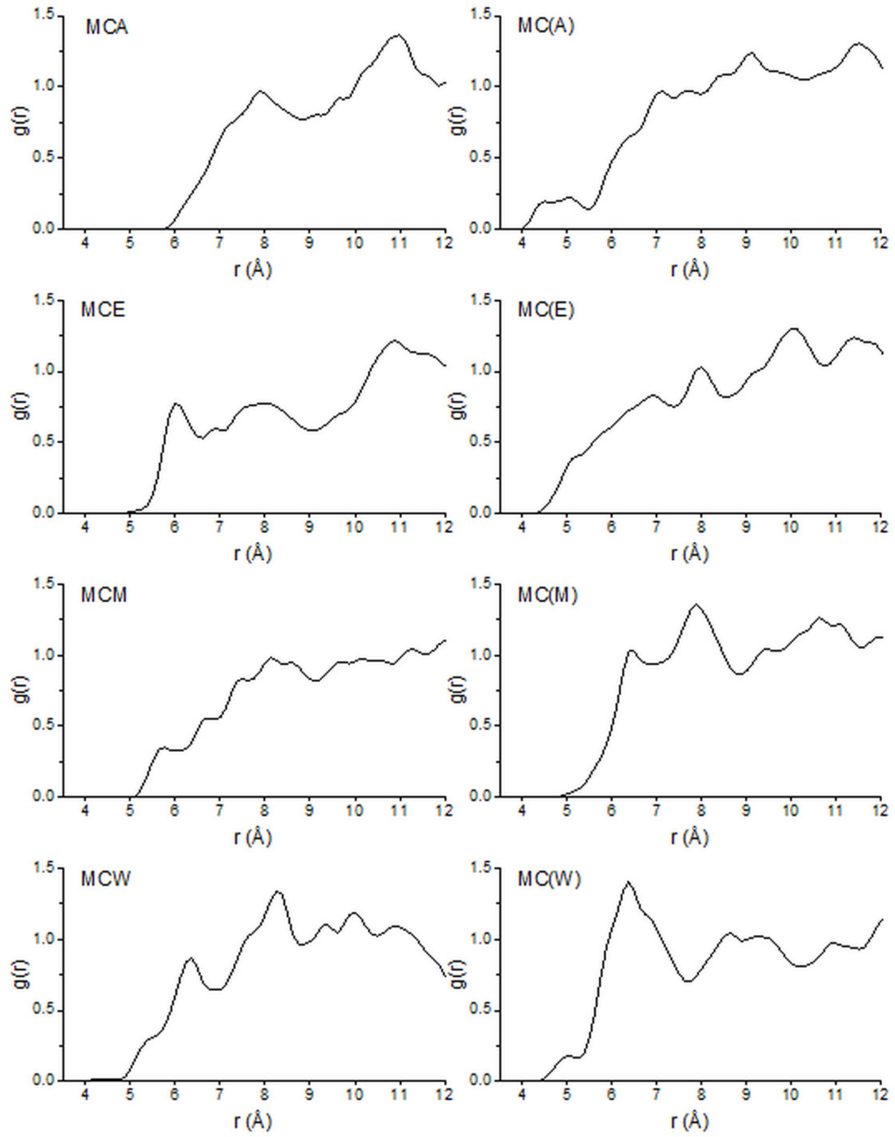

Figure S5. The Radial Distribution Functions of the centers of the MC molecules for solvated and de-solvated MC molecules (MCX - solvated MC with the solvent X=A, E, M, W, and MC(X) – de-solvated MCX).

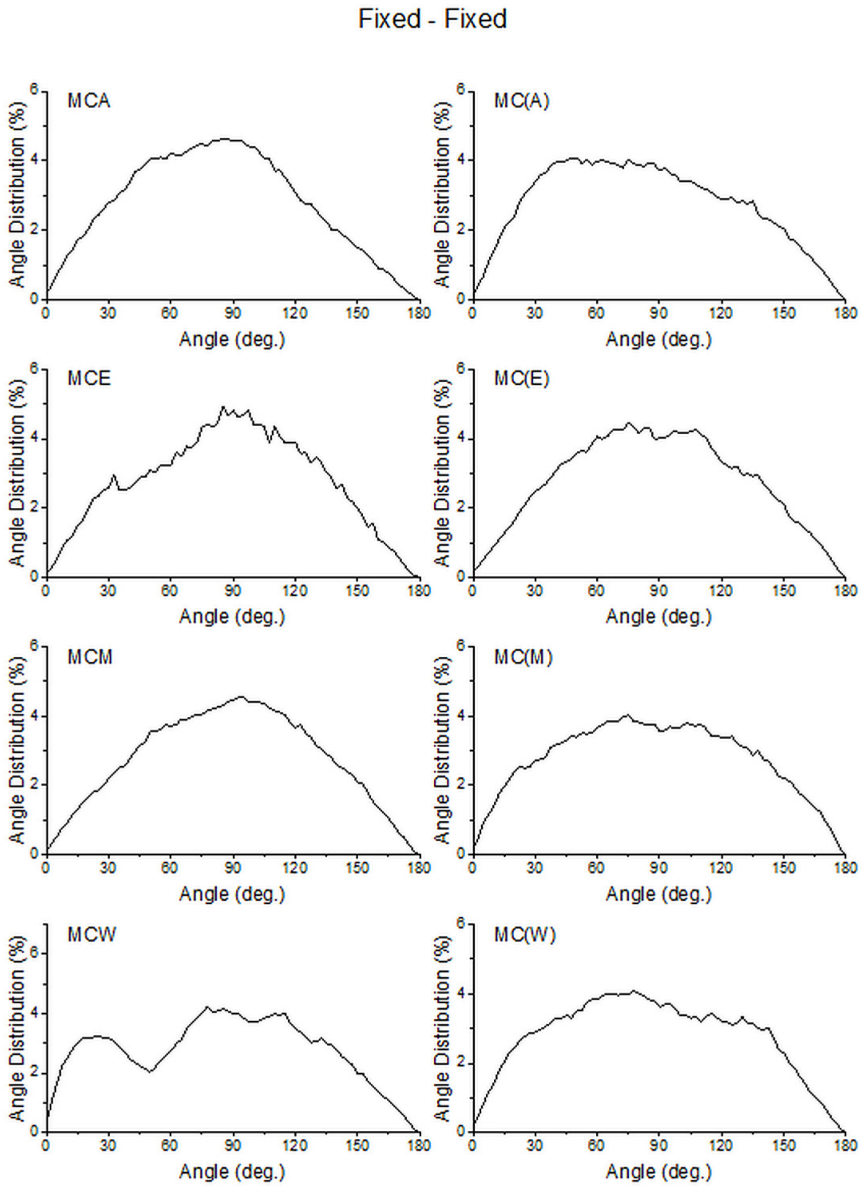

Figure S6. The distributions of angles formed by normal vectors to the average planes that contain the fixed fragments of two neighbor MC molecules for solvated (left column) and de-solvated (right column) MC molecules (MCX - solvated MC with the solvent X=A, E, M, W, and MC(X) – de-solvated MCX).

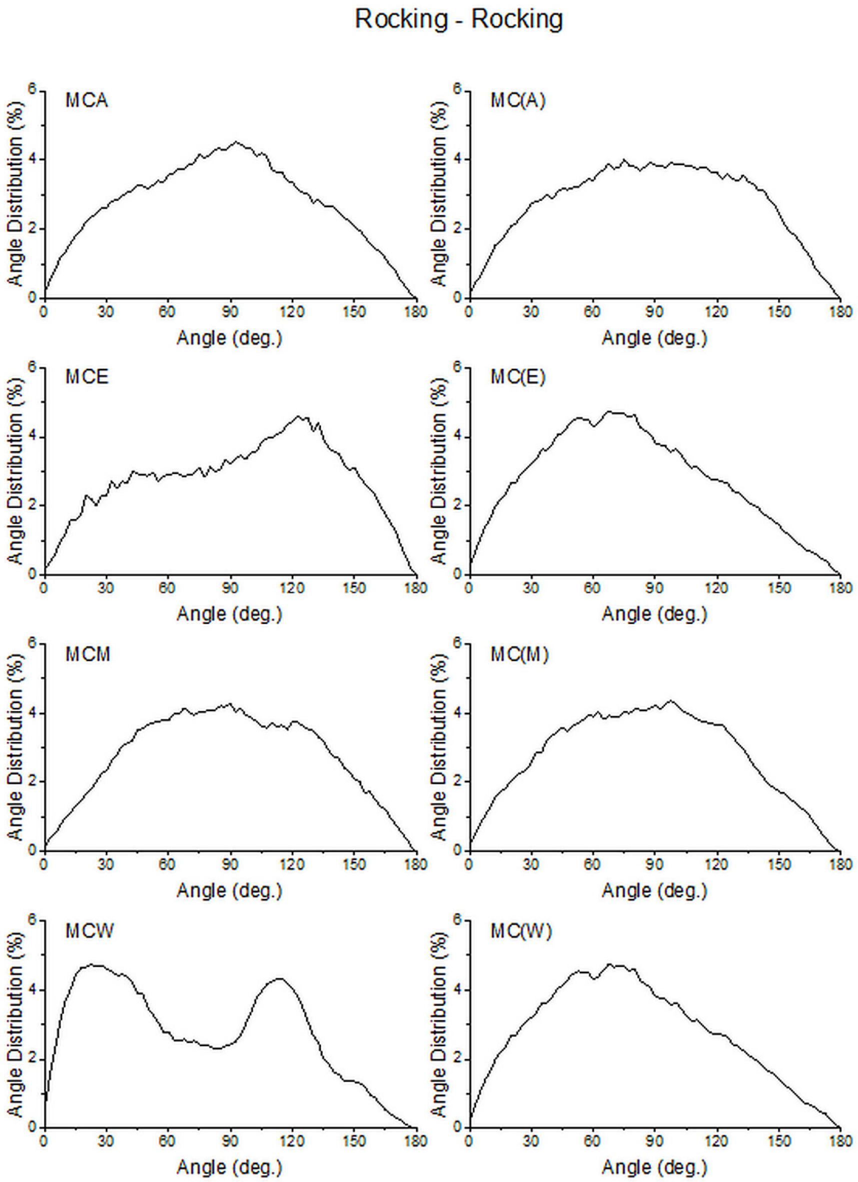

Figure S7. The distributions of angles formed by normal vectors to the average planes that contain the rocking fragments of two neighbor MC molecules for solvated (left column) and de-solvated (right column) MC molecules (MCX - solvated MC with the solvent X=A, E, M, W, and MC(X) – de-solvated MCX).

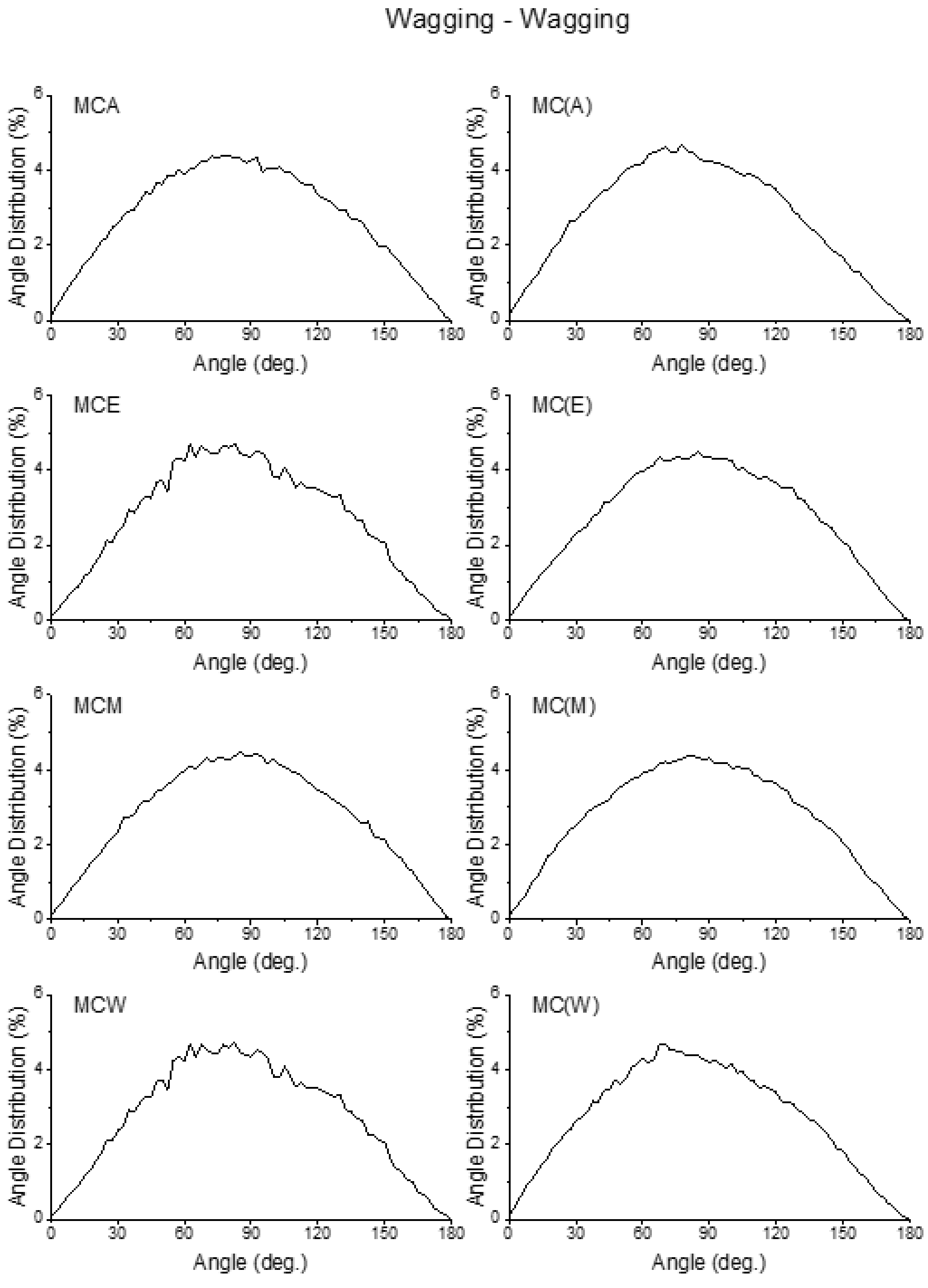

Figure S8. The distributions of angles formed by the orientation vectors of the wagging fragments of two neighbor MC molecules for solvated (left column) and de-solvated (right column) MC molecules (MCX - solvated MC with the solvent X=A, E, M, W, and MC(X) – de-solvated MCX).

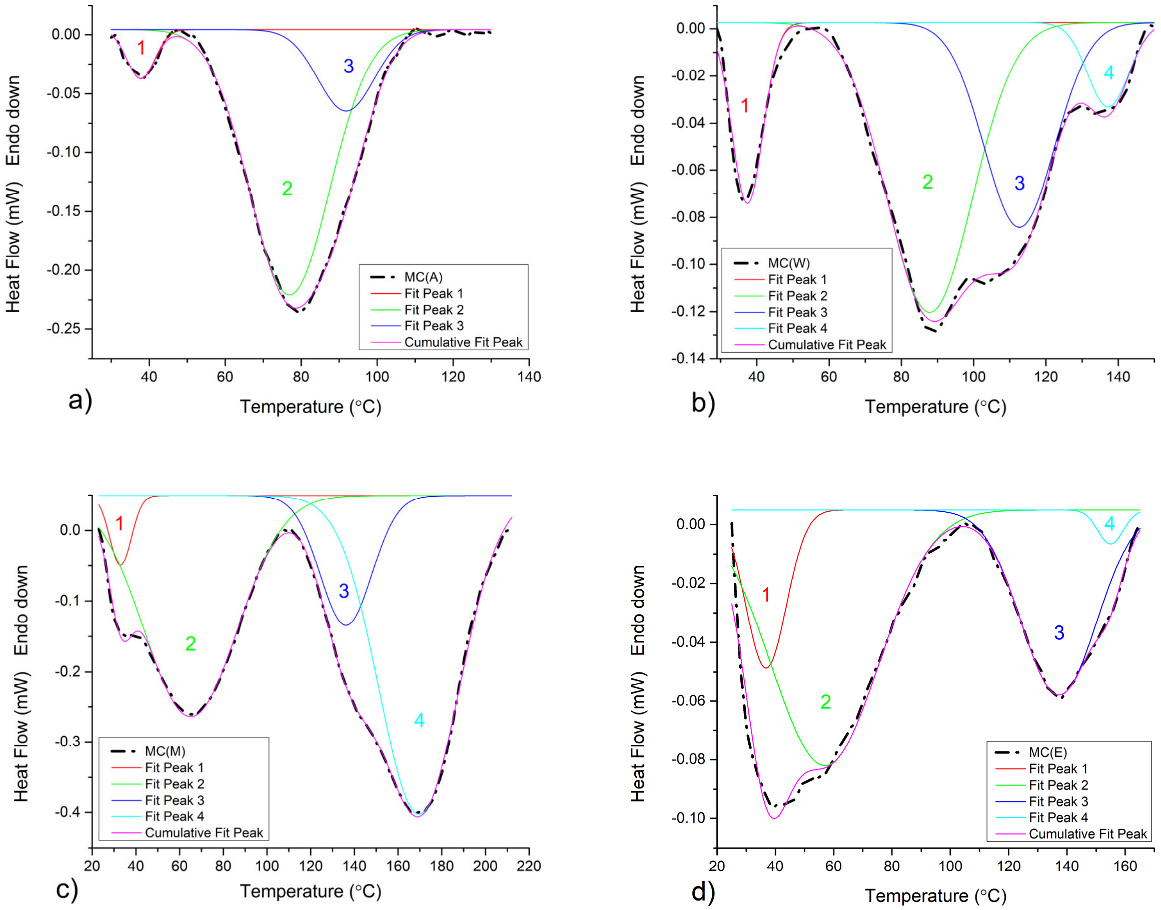

Figure S9. DSC curves for MC(A) - (a), MC(W) - (b), MCM - (c) and MC(E) - (d). The curves are de-convoluted using Gaussian distributions. The magenta line following the experimental curve (black dash-dot line) represents the fitted DSC curve. For each of the peaks, labelled as 1-4 (as the temperature increases), the temperature corresponding to the peak and fractional areas are given in Table S2.

Table S2. The values and standard deviations of the temperatures *T* corresponding to the peaks in the DSC scans in Figure S9, as well as the corresponding fractional areas, *A*, for the assigned components (1-4) of the DSC curves in different MC de-solvates (from Figure S9). The coefficients of determination in all cases are $R^2 > 0.998$.

Sample	T^1 [C]	T^2 [C]	T^3 [C]	T^4 [C]	A^1 %	A^2 %	A^3 %	A^4 %
MC(A)	37.8±0.02	76.9±0.89	91.8±0.21	-	1.9±1.22	85.2±1.78	12.9±5.66	-
MC(W)	37.4±0.02	72.5±0.32	86.5±0.14	137.7±0.09	5.0±0.89	61.4±1.22	29.5±1.49	4.1±1.49
MC(M)	32.9±0.04	65.1±0.04	136.2±0.11	169.6±0.07	0.8±0.67	44.2±0.50	7.9±0.85	47.1±0.45
MC(E)	36.8±0.07	56.9±0.45	137.3±0.21	154.9±0.28	6.1±5.74	68.8±2.73	24.6±2.43	0.5±9.26
